# Predicting the risk of acute kidney injury after cardiopulmonary bypass: development and assessment of a new predictive nomogram

**DOI:** 10.1186/s12871-022-01925-w

**Published:** 2022-12-07

**Authors:** Huan Jing, Meijuan Liao, Simin Tang, Sen Lin, Li Ye, Jiying Zhong, Hanbin Wang, Jun Zhou

**Affiliations:** 1 The Third Affiliated Hospital of Southern Medical University, 183 Zhongshan Avenue West, Tianhe District, Guangdong Province Guangzhou City, China; 2 The First People’s Hospital of Foshan, 81 Lingnan Avenue, Chancheng District, Guangdong Province Foshan City, China

**Keywords:** Cardiopulmonary bypass, Acute kidney injury, Prevention, Cardiac surgery, Nomogram

## Abstract

**Background:**

Acute kidney injury (AKI) is a common and severe complication of cardiac surgery with cardiopulmonary bypass (CPB). This study aimed to establish a model to predict the probability of postoperative AKI in patients undergoing cardiac surgery with CPB.

**Methods:**

We conducted a retrospective, multicenter study to analyze 1082 patients undergoing cardiac surgery under CPB. The least absolute shrinkage and selection operator regression model was used to optimize feature selection for the AKI model. Multivariable logistic regression analysis was applied to build a prediction model incorporating the feature selected in the previously mentioned model. Finally, we used multiple methods to evaluate the accuracy and clinical applicability of the model.

**Results:**

Age, gender, hypertension, CPB duration, intraoperative 5% bicarbonate solution and red blood cell transfusion, urine volume were identified as important factors. Then, these risk factors were created into nomogram to predict the incidence of AKI after cardiac surgery under CPB.

**Conclusion:**

We developed a nomogram to predict the incidence of AKI after cardiac surgery. This model can be used as a reference tool for evaluating early medical intervention to prevent postoperative AKI.

**Supplementary Information:**

The online version contains supplementary material available at 10.1186/s12871-022-01925-w.

## Introduction

Acute kidney injury (AKI) is a common and severe complication of cardiac surgery with CPB [1, 2]. The apparent characteristics of AKI are rapid development, poor prognosis, high mortality, and complicated pathogenesis [3–5]. Studies have shown that as many as 42% of patients undergoing CPB heart surgery have cardiac surgery-related AKI after surgery, of which 2–6% of patients require continuous renal replacement therapy (CRRT), and the associated survival time is reduced, which usually bring a heavy burden to society. At present, there are few effective treatments for CPB-related AKI in the world, mainly including conservative medication and CRRT as supportive measures [6, 7]. And clinically, the diagnosis of AKI is mainly based on serum creatinine (Scr) and urea nitrogen levels, but these indicators are not sensitive to the early prediction of kidney damage [8, 9]. Furthermore, an emerging method in recent years is to detect biomarkers such as NGAL, KIM-1, miRNA-21 in blood or urine for early diagnosis of AKI. Whereas, these biomarkers are usually only a few hours earlier than the detection of Scr, and some biomarkers are also present in other diseases and cause false positives [10]. Thereby, it is necessary to identify high-risk patients early and give appropriate postoperative AKI support.

In order to rationally manage AKI after cardiac surgery, a model that can accurately predict high-risk patients to optimize postoperative AKI treatment strategies is very urgent. The Cleveland University Acute Renal Failure Score System (Cleveland Score) [11], Simplified Kidney Index Score (SRI Score) and other researchers have established predictive models of AKI after cardiac surgery, which are widely used [12]. However, these models are mainly based on old clinical data collected more than ten years ago, and mainly come from western populations. For clinicians, early prediction, early diagnosis and management of AKI after cardiac surgery are still challenging. Hence, the establishment of risk models for such patients still has important clinical significance.

The nomogram transforms the complex regression equation into a visual graph, making the results of the prediction model easier to read and more convenient to evaluate the patient’s condition. Due to its intuitive and easy-to-understand characteristics, nomogram has gradually received more and more attention and applications in medical research and clinical practice [13, 14].

However, nomograms are rarely used in AKI after cardiac surgery. Therefore, this study intends to use perioperative clinical information to include some preoperative and intraoperative risk factors that may affect the occurrence of postoperative AKI into the study. According to the AKI standard proposed by KDIGO in 2012, a nomogram prediction model was established through multivariate logistic regression analysis. Predict the prevalence of AKI in patients undergoing CPB cardiac surgery, and provide clinical guidance for early identification and intervention of high-risk patients [15].

## Materials and methods

### Study population

A retrospective analysis (2015–2020) of patients > = 18 years of age who underwent CPB cardiac surgery collected from two Grade A hospitals in China (Foshan First People’s Hospital; Zhongshan People’s Hospital). Excluding long-term dialysis patients with chronic renal failure and patients with incomplete clinical data, a total of 1082 patients were included in the study. 758 patients assigned to the modeling group and 324 assigned to the verification group. In addition, a total of 108 patients who met the inclusion criteria in Guangzhou First People’s Hospital from January 1, 2020 to October 1, 2020 were retrospectively collected. These 108 patients were used as an external validation group to verify the effectiveness of the model.

### Data collection and definitions

The risk factors for preoperative AKI included the patient’s age, sex, and pre-surgical history of hypertension, diabetes, or anemia. We investigated CPB time, blood transfusion volume, urine volume, and the use of vasoactive and other drugs as intraoperative variables.

According to the clinical practice guidelines for AKI in Kidney Disease: Improving Global Outcomes (KDIGO), AKI is defined as any of the following: an increase in Scr of 0.3 mg/dl (26.5µmol/L) within 48 h or Scr increased to 1.5 times the baseline level within 7 days, or urine volume < 0.5 ml/kg/h for 6 h [16]. In this study, we used blood creatinine values to define acute kidney injury. Baseline Scr levels were defined as the values obtained closest to the date of surgery.

### Statistical analysis

Statistical analyses were performed using the R software (Version 3.6.2; https://www.R-project.org) and SPSS21.0 software. First, the minimal absolute contraction and selection operator (LASSO) method for reducing high-dimensional data was used to select the best predictive characteristics of risk factors for postoperative AKI in patients who had undergone cardiac surgery with CPB [17, 18]. Select variables with non-zero coefficients in the LASSO regression model. Subsequently, through multivariate logistic regression analysis, the variables with *p*-value < 0.05 were used to construct a prediction model. Harrell’s C index was measured to quantify the discriminative performance of the nomogram [19]. Following this, calibration curves were plotted to assess the calibration of the nomogram. We analyzed the decision curve to determine the clinical effectiveness of the nomogram by quantifying the net benefits under different threshold probabilities in the cohort. Moreover, internal verification is performed by calculating the C index of the verification group. Finally, we included the variables included in the logistic regression model, reconstructed the model using a neural network, and evaluated the variable importance and model performance.

## Results

### Patient characteristics

We divided the 1082 patients included in this study into AKI group (288) and non-AKI group (794) according to KDIGO standards. Table 1 shows the preoperative and intraoperative factors of the two groups of patients. 70% (758) patients were randomly selected to establish the prediction model, and 30% (324) patients were used to verify the accuracy of the model.

**Table 1:** Differences between influencing factors of AKI and non-AKI groups

Influencing factors	AKI, *n*=288	Non-AKI, *n*=794	Total, *n*=1082
**Preoperative factors**			
Age	57 (49, 66)	51 (43, 62)	54 (45, 63)
Sex			
Male	202 (70.1%)	388 (48.9%)	590 (54.5%)
Female	86 (29.9%)	406 (51.1%)	492 (45.5%)
Diabetes			
Yes	20 (6.9%)	32 (4.0%)	52 (4.8%)
No	268 (93.1%)	762 (96.0%)	1030 (95.2%)
Hypertension			
Yes	99 (34.4%)	130 (16.4%)	229 (21.2%)
No	189 (65.6%)	664 (83.6%)	853 (78.8%)
Anemia			
Yes	93 (32.3%)	205 (25.8%)	298 (27.5%)
No	195 (67.7%)	589 (74.2%)	784 (72.5%)
**Intraoperative factors**			
CPB time	186 (122, 230)	161 (105, 203)	168 (110, 208)
RBC	5.5 (1.5, 7)	3.6 (0, 5.5)	4.1 (0, 6)
Plasma	460 (0, 600)	321 (0, 600)	358 (0, 600)
Cryoprecipitate	4 (0, 0)	2.3 (0, 0)	2.7 (0, 0)
Albumin	100 (20, 100)	95 (20, 100)	96 (20, 100)
Voluven^®^	376 (0, 500)	391 (0, 500)	387 (0, 500)
Gelofusine^®^	354 (0, 768)	232 (0, 500)	264 (0, 500)
Equilibrium liquid	2156 (1425, 2500)	2095 (1500, 2500)	2111 (1500, 2500)
5% SBS	70 (0, 90)	32 (0, 0)	42 (0, 0)
Furosemide	8 (0, 20)	7 (0, 15)	7 (0, 15)
Intraoperative blood loss	894 (300, 800)	565 (300, 600)	653 (300, 600)
Urine	1436 (600, 2000)	1485 (800, 2000)	1472 (800, 2000)

### Feature selection

**Fig. 1:**
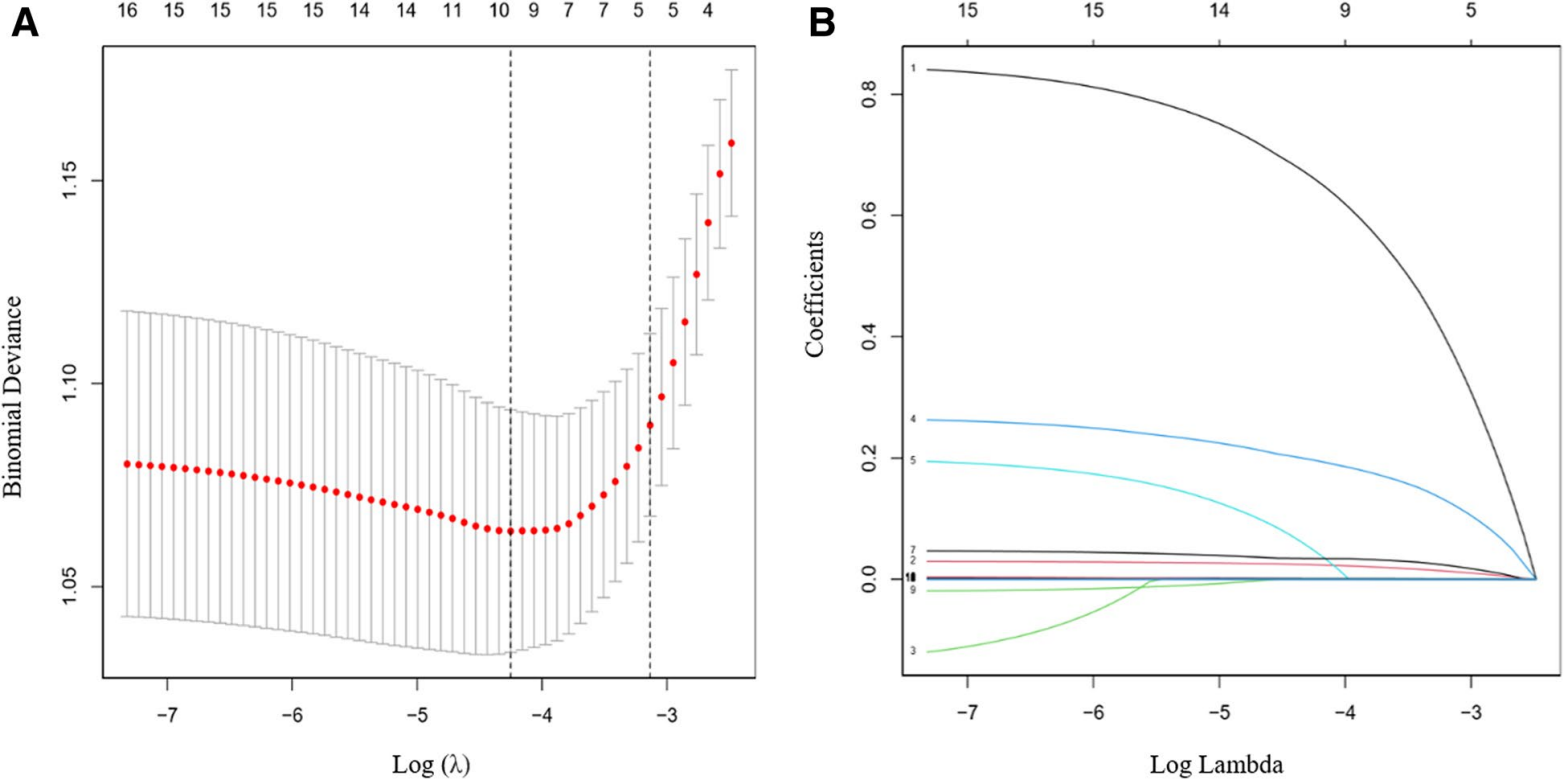
The LASSO binary logistic regression model was used to select the risk factors of AKI after CPB. **A** The choice of the best parameter (lambda) in the LASSO model passed the lowest standard for five times of cross-validation. The partial likelihood deviation (binomial deviation) curve is plotted against log (λ). Draw a dashed vertical line with the best value by using the minimum standard and the minimum standard 1 SE (1-SE standard). **B** LASSO coefficient curve of 17 features. A coefficient distribution map for the logλ sequence is generated. Vertical lines are drawn at the values selected using five-fold cross-validation, where the optimal λ produces sixteen features with non-zero coefficients

In terms of preoperative and intraoperative influencing factors, according to the validation group of 758 patients (~ 2:1 ratio; Fig. 1 A and Fig. 1B), 17 variables were reduced to 10 potential predictors, and they were returned in LASSO. There are non-zero coefficients in the model. These variables include age, sex, hypertension, anemia, CPB duration, intraoperative succinylated gelatin and 5% bicarbonate solution infusion (SBS), as well as red blood cell (RBC) infusion, intraoperative blood loss and urine output (Table 2).

**Table 2:** Predictive factors of acute kidney injury in cardiac surgery after CPB

Variables	Multivariable analysis		
	OR	95%CI	P
Age	1.028	1.013,1.043	0.000
Sex	2.026	1.407,2.917	0.000
Hypertension	2.010	1.338,3.019	0.001
CPB	1.003	1.000,1.005	0.024
RBC	1.064	1.024,1.105	0.001
5% SBS	1.002	1.000,1.004	0.015
Urine	1.000	1.000,1.000	0.023

### Predictive nomogram for the probability of AKI

**Fig. 2:**
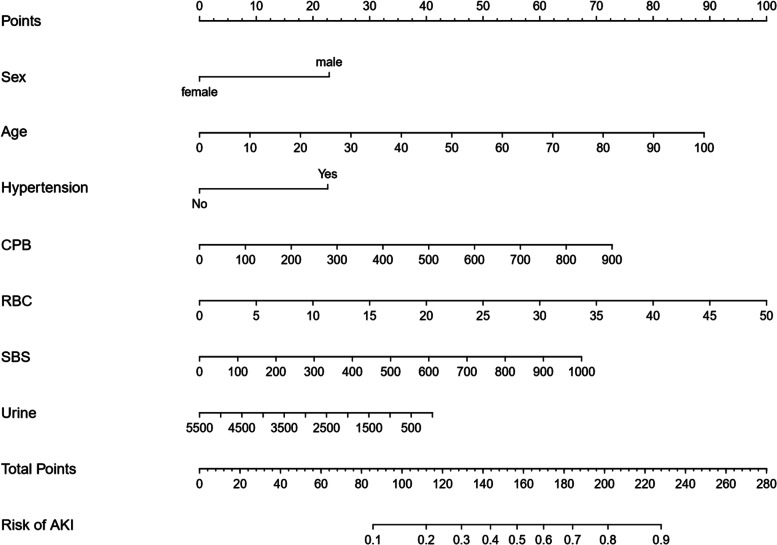
The nomogram of AKI after CPB surgery. The Nomogram of AKI after CPB surgery estimates the probability of AKI after the operation. Mark the patient value on each axis, draw a straight line perpendicular to the point axis, and add the points of each variable. Next, mark the sum on the total point axis and draw a straight line perpendicular to the probability axis. RBC- intraoperative transfusion of red blood cells; SBS-intraoperative 5% sodium bicarbonate solution infusion; Urine-intraoperative urine output

Based on the final regression analysis, a nomogram containing seven essential risk factors for predicting AKI in patients who had undergone cardiac surgery with CPB was constructed. The total score was calculated based on gender, age, hypertension, CPB duration, intraoperative 5% SBS and RBC transfusion, and urine output. The value of each variable was assigned a score on the point scale axis. The total score could be calculated easily based on addition of individual scores. Following this, the overall score was projected into a total score (Fig. 2). In this manner, we could estimate the probability of AKI in patients who had undergone cardiac surgery with CPB.

### Apparent performance of the AKI risk nomogram in the cohort

**Fig. 3:**
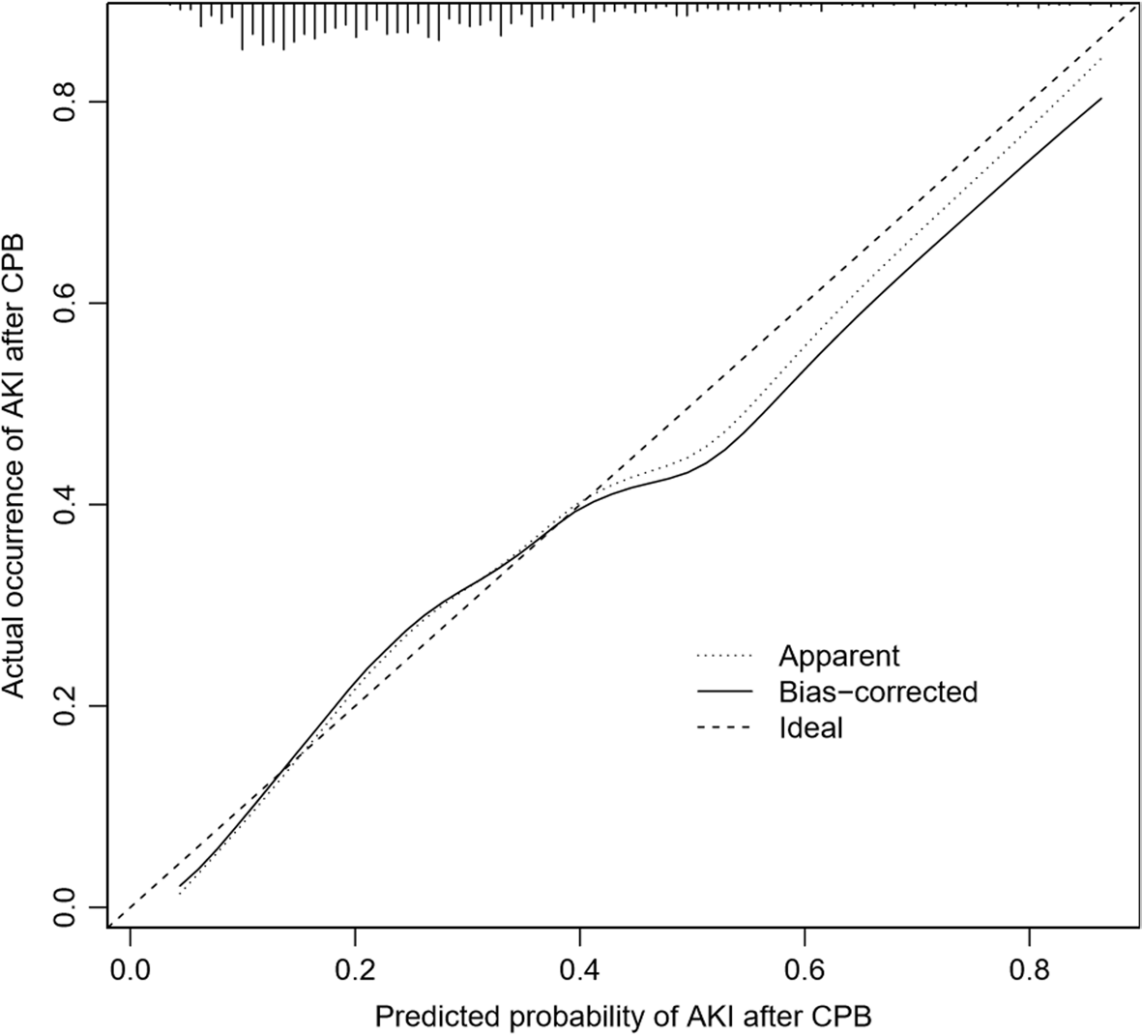
Calibration curve for nomogram prediction of AKI after CPB in the cohort. The x-axis represents the predicted risk of AKI after CPB. The y-axis represents the actual occurrence of AKI after CPB. The dotted line on the diagonal represents the perfect prediction of the ideal model. The solid line represents the performance of the nomogram. The closer the fit to the diagonal dashed line is, the better the prediction effect

The calibration curve of the nomograms used to predict the risk of AKI in patients after surgery showed good agreement (Fig. 3). The C index of the predicted nomogram of this group was 0.725 (95% confidence interval: 0.685–0.765). Furthermore, the value was confirmed as 0.709 by the verification group, which indicated good degree of recognition of the model. In order to further verify the effectiveness of the model, we also retrospectively collected 108 patients who met the inclusion criteria in Guangzhou First People’s Hospital from January 1, 2020 to August 1, 2020 as an external validation. The median age of the external validation group was 58.5 years (50, 66), the proportion of male patients was 35.2%, the preoperative hypertension patients were 27.8%, the median CPB time was 94.5 min (60, 135), and the median red blood cell transfusion volume was 2u (0, 4.5), the median intraoperative sodium bicarbonate injection infusion was 0 ml (0, 0), and the median urine output was 800 ml (500, 1000). According to the establishment of the above model, the C-index of the external validation group is 0.973.

### Clinical applications

**Fig. 4:**
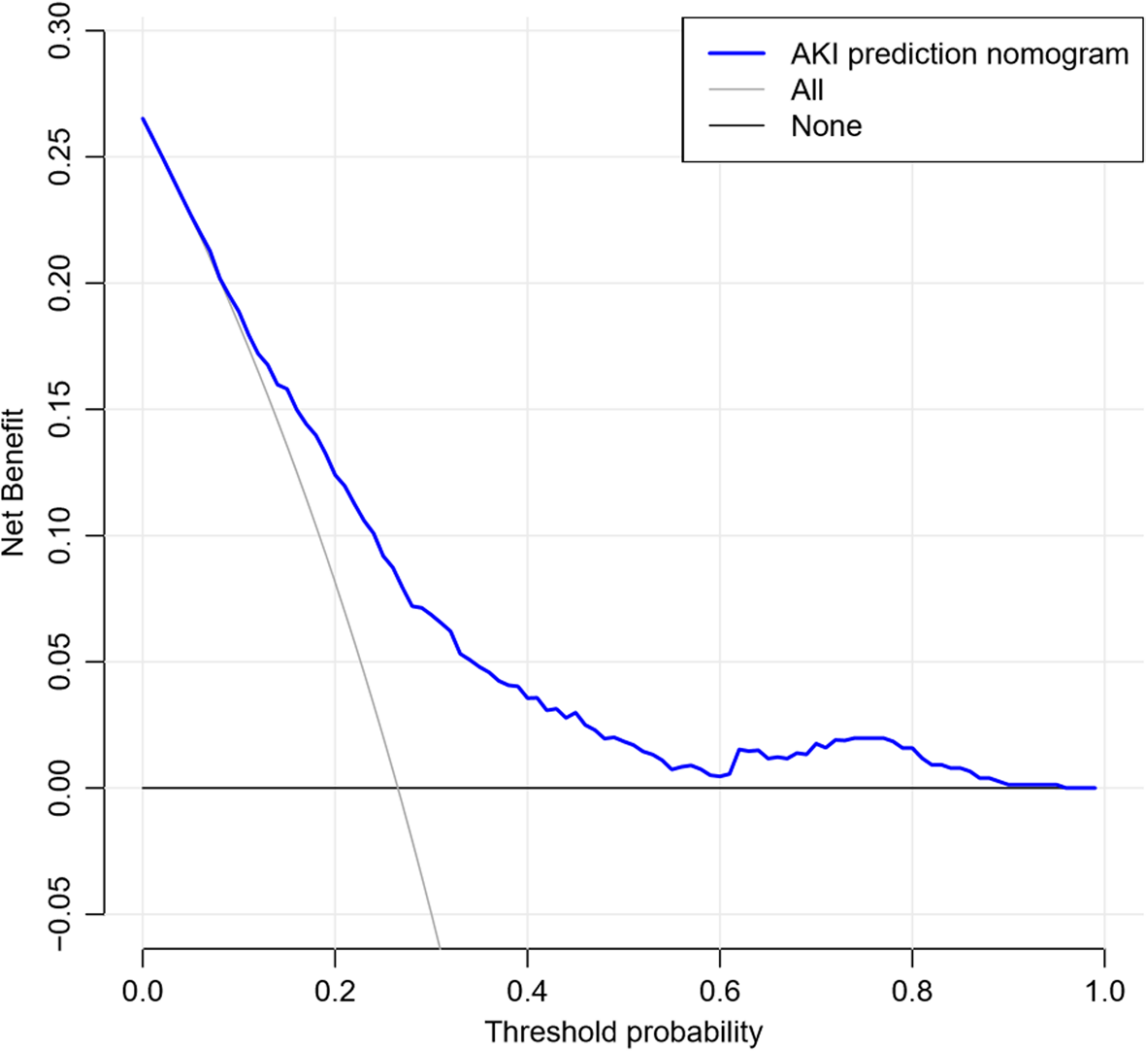
Decision curve analysis for the AKI nomogram. The y-axis is net income. The blue line represents the distribution of AKI after CPB. The thin solid line indicates that it is assumed that all patients undergoing CPB surgery do not develop AKI after surgery. The thick solid line represents the hypothesis that all patients have AKI. The decision curve shows that if the threshold probability is greater than 4%, the use of the nomogram of AKI after CPB in the current study to predict the risk of AKI will increase the benefit more than the full-patient intervention program or the non-intervention program

Analysis of the decision curve of the AKI nomogram is shown in Fig. 4. The decision curve shows that if the threshold probability is greater than 4%, the use of the nomogram of AKI after CPB in the current study to predict the risk of AKI will increase the benefit more than the full-patient intervention program or the non-intervention program.

### Neural network validation

**Fig. 5:**
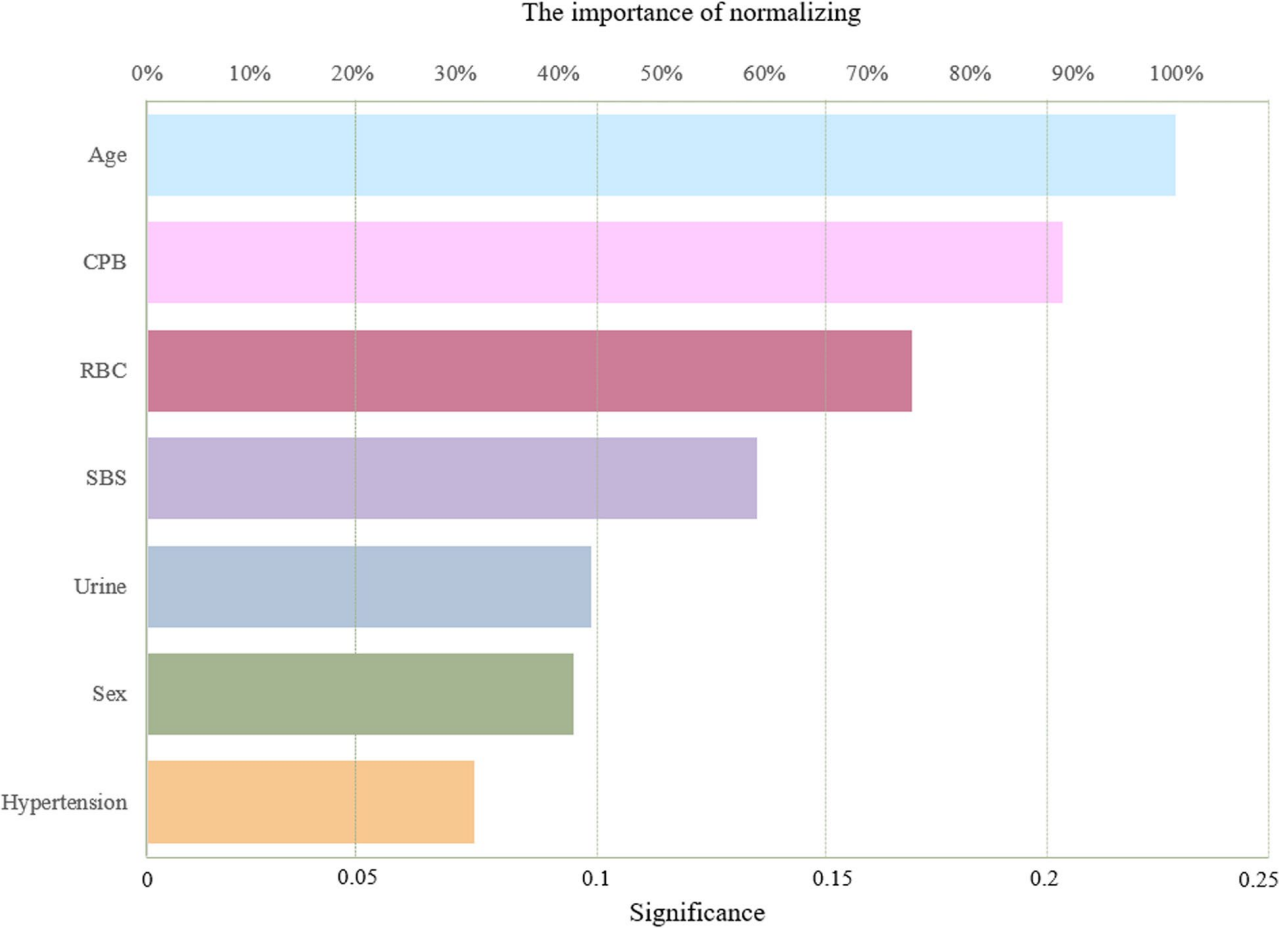
The importance of normalizing. The independent variable importance map can be used to analyze which factors have a greater impact on the predicted value, and the more obvious the importance, the greater the impact on the predicted value. This figure suggests that Age has the greatest impact on postoperative AKI.

**Fig. 6:**
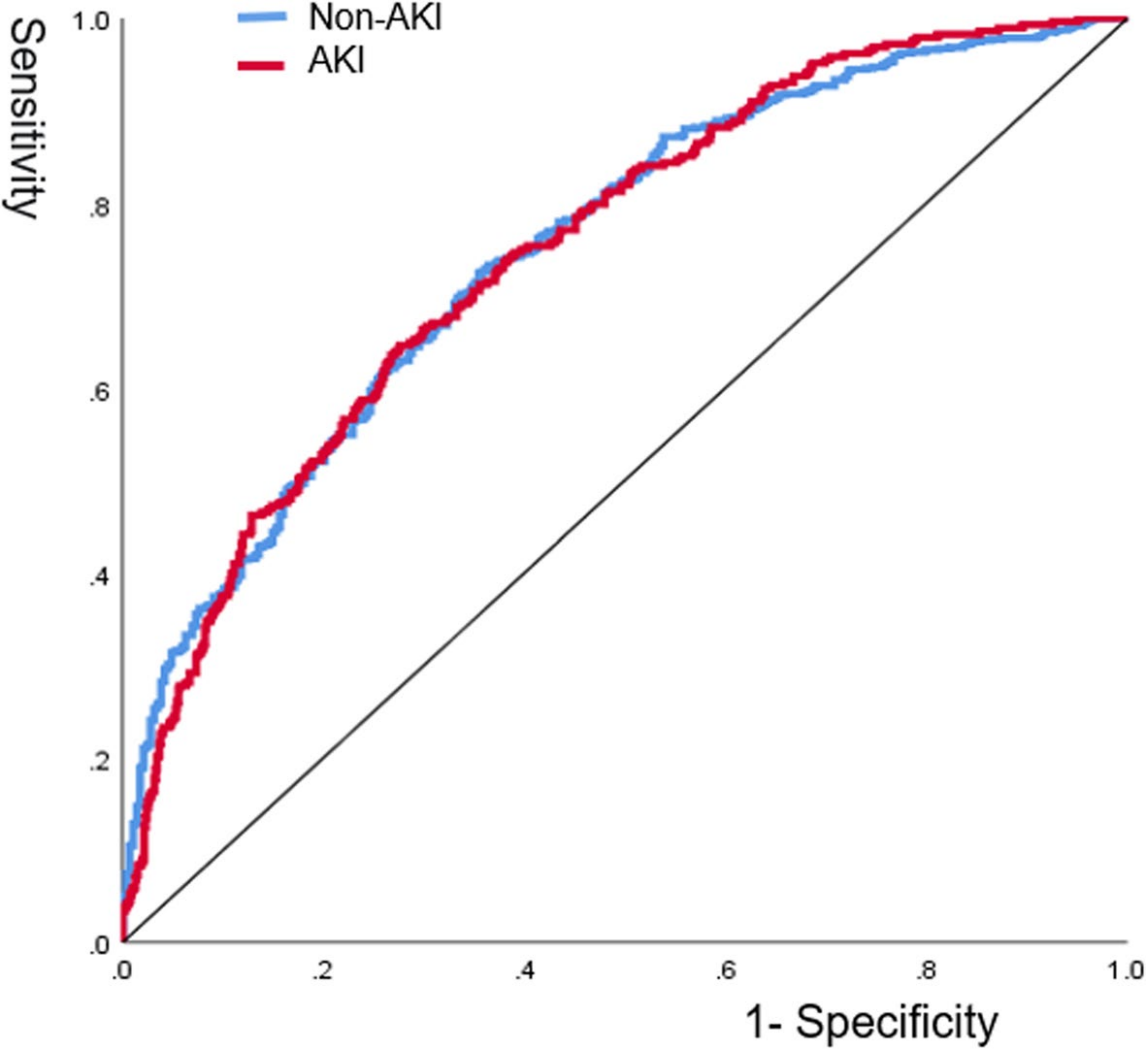
ROC curve of neural network model. The ROC curve is a curve reflecting the relationship between sensitivity and specificity. The abscissa (X-axis) is 1 – specificity, also known as the false positive rate (false positive rate), the closer the X-axis is to zero, the higher the accuracy; the ordinate (Y-axis) is called sensitivity, also known as true positives rate (sensitivity), the larger the Y-axis, the better the accuracy. The area under the ROC curve (AUC) can evaluate the quality of the model. If the area under the ROC curve is greater than 0.5, it proves that the model has certain value. The closer the AUC is to 1, the better the authenticity of the model is proved. The area under the ROC curve of this neural network model was 0.749, which confirmed the good value of this model

Artificial Neural Network (ANN) is a statistical method with similar characteristics generated by people simulating the structure and function of the human brain [20]. Logistic regression and ANN have essential differences. Although both are popular algorithms in the field of machine learning, they have different advantages in different aspects: Compared with the readability of the model, Logistic regression has a great advantage over neural network. In most cases, because of the complex internal operation mechanism of neural network, the output results are directly obtained from the input, and people can use the knowledge induced by the model without understanding its operation [21]. We reconstruct the neural network model with the independent variables included in the above logistic regression model. First, we used a multilayer perceptron to perform neural network model with postoperative AKI as the dependent variable and gender, age, hypertension, CPB time, intraoperative red blood cell transfusion, intraoperative SBS transfusion, and urine volume as covariates. The 1082 patient data were randomly split into 761 training sets (70.3%) and 321 validation sets (29.7%). In addition, the function of model building activation is a hyperbolic tangent function, and the weights are updated using the scaled conjugate gradient method. The model confusion matrix is shown in Table 3. Next, the importance of independent variables is evaluated according to the results of feature importance analysis of the neural network model, and the ranking is shown in Fig. 5; Table 4. The results showed that the order of importance of independent variables was age, CPB time, RBC infusion, SBS infusion, urine output, gender, and hypertension. We unexpectedly found that the results were consistent with the order of axis lengths represented by the independent variables of the above nomogram. Finally, we evaluate the performance of the neural network model. The ROC curve of the model is shown in Fig. 6, and the area under the curve is 0.749, which indicates that the model has good generalization ability.

**Table 3:** Neural network model confusion matrix

Sample	Measured	Prediction		
		Non-AKI	AKI	Accuracy (%)
Training set	Non-AKI	516	38	93.1%
	AKI	147	58	28.3%
	Overall percentage	87.4%	12.6%	75.6%
Testing set	Non-AKI	224	16	93.3%
	AKI	57	26	31.3%
	Overall percentage	87.0%	13.0%	77.4%

**Table 4:** Importance of independent variables

	Significance	Standardized importance
Sex	0.111	49.2%
Age	0.216	96.1%
Hypertension	0.135	60.0%
CPB	0.134	59.8%
RBC	0.225	100.0%
SBS	0.118	52.6%
Urine	0.061	27.4%

## Discussion

In 1953, the successful implementation of the first open heart surgery under CPB officially opened the prelude to cardiac surgery. This is considered to be one of the most important clinical developments in the medical field [22]. The CPB technology temporarily replaces the human heart and lungs with an external circuit composed of a pump and an oxygenation membrane. Its purpose is to replace the heart and lung function to maintain the blood perfusion of the whole body tissues and organs when performing heart surgery, and at the same time create a good surgical field of vision for the surgeon [23]. Although CPB has now become an indispensable technique for many heart operations, the potential adverse effects of this technique on sensitive organs such as the brain or kidney cannot be ignored. During CPB, patients are exposed to a complex set of non-physiological conditions. Blood coagulation, pro-inflammatory, activation of survival cascade and changes in redox state can all trigger systemic inflammatory response syndrome, leading to multiple organ dysfunction [24]. Compared to other specialized surgeries, the operative morbidity and mortality of patients undergoing cardiac surgery with CPB are higher. The incidence of postoperative complications has been of great concern [25]. As a common clinical complication of cardiac surgery, AKI significantly reduces the cure rate and quality of life of the patients [26, 27]. We observed that AKI occurred in approximately 27% of the patients in our study. In the analysis of risk factors, postoperative AKI was related to gender, age, hypertension, CPB duration, intraoperative 5% SBS and RBC transfusion, and urine output. The nomogram shows that males, older age, preoperative hypertension, longer CPB duration, intraoperative RBC and 5% SBS infusion, and less urine output will increase the risk of AKI after surgery. Hence, it is necessary and meaningful to take preventive measures. The current clinical diagnosis of AKI chiefly relies on assessment of Scr and urea nitrogen levels [28]. However, these traditional biomarkers do not enable early referral to clinicians, thus preventing early intervention to avoid the occurrence of AKI. Therefore, an accurate prediction method of postoperative AKI in patients undergoing cardiac surgery with CPB is clinically crucial.

In the present study, we built a model to predict the risk factors of AKI in patients who underwent cardiac surgery with CPB. The nomogram, which transforms the statistical prediction model into a simple and intuitive graph, so as to calculate the numerical probability of the occurrence of clinical events. The nomogram first builds a multi-factor regression model, assigns points to each value level of each influencing factor, and adds up the points of each segment to obtain the total score. Finally, the total score and the probability of an outcome event are converted by a function to obtain the predicted probability of an individual outcome event. We specifically studied the relationship between preoperative and intraoperative influencing factors and postoperative AKI. Internal validation of the cohort showed good discrimination and calibration capability. In particular, the high C index value in the interval validation indicated that the collinear map could be used widely and accurately for large samples.

Each factor of the nomogram represents the length of the line segment, which reflects the contribution of the factor to the end event. Sexual dimorphism has been correlated with AKI. Large-scale epidemiological studies have shown that female have a lower incidence of AKI after non-cardiac surgery. Interestingly, based on various retrospective and prospective studies, national and international registration data, most researches on AKI after cardiac surgery consider female to be an independent risk factor for its development [29–31]. Nevertheless, our findings contradict the widely accepted view that female are an independent risk factor for the development of AKI after cardiac surgery. In 2016, Neugarten et al. also reached a contradictory conclusion through meta-analysis, they think this may simply reflect a different definition of AKI after heart surgery [32]. Whereas, this simple explanation may obscure the more complex interaction between gender and cardiovascular surgery. Furthermore, previous studies on animal models have reported that female sex had an inherent protective role against ischemia reperfusion induced AKI, and testosterone increases the susceptibility of the kidney to ischemic injury [9, 33–35]. The relationship between gender and AKI after cardiac surgery still needs further research.

Recent studies have shown that with age, the structure and function of the kidney undergo characteristic changes. These changes impair the ability of the kidney to withstand and recover from injury, resulting in a high sensitivity of the elderly to AKI [36]. Previous studies have shown that nephrons are stably lost, and the glomerular filtration rate decreases with age (in individuals aged ≥ 30 years) [37]. Moreover, a large number of clinical studies have also confirmed that the elderly are more likely to develop AKI after cardiac surgery and have a worse prognosis than young patients [38]. Similarly, our study confirmed that age is an independent risk factor for AKI after cardiac surgery with CPB.

The relationship between kidneys and hypertension has been extensively studied. Persistent high blood pressure will increase the hydrostatic pressure of the renal blood vessels and cause thickening of the arterial wall. This is due to the increase in the smooth muscle and hyaluronic acid content of the medium and the narrowing of the lumen, which in turn leads to a decrease in renal blood flow. In CPB heart surgery, the kidney is in a state of hypoperfusion, these changes also make the kidney more prone to prerenal AKI [39]. Studies have confirmed that preoperative hypertension is an independent risk factor for AKI after CPB. They believe that long-term persistent hypertension can increase the pressure of the glomerulus, leading to pathological changes such as glomerular fibrosis and vascular sclerosis. Even if the renal function is normal before surgery, there may be renal parenchymal ischemia and nephron reduction. This will increase the risk of AKI after CPB. In 2018, Barkhordari et al. conducted a cross-sectional study of 3473 patients, and the results showed that hypertension was significantly associated with the incidence of AKI after coronary artery bypass graft surgery [40]. The results of this study indicate that patients diagnosed with hypertension before surgery have an increased risk of AKI after CPB, which is consistent with most studies.

Acute blood loss or decreased hematocrit is common in heart surgery, so blood transfusion is often performed during surgery to improve oxygen supply to the kidneys and other important organs [41]. Throughout the ages, restrictive blood transfusion and liberal blood transfusion programs have been controversial and have been widely studied. However, studies have confirmed that blood transfusion is not a benign intervention and has been associated with multiple organ failure, including AKI in patients undergoing CPB. Recently, data from a randomized controlled trial showed that in patients undergoing cardiac surgery, restrictive blood transfusion regimens are not inferior to free blood transfusion regimens [42]. Rasmussen et al. confirmed that the transfusion of all blood products increased the risk of AKI in a dose-dependent manner. However, in a multivariate analysis combining all blood products, only red blood cell transfusion is still significantly associated with the occurrence of acute kidney injury [43]. This is consistent with our conclusion. At present, several pathophysiological mechanisms of kidney injury after red blood cell infusion have been proposed. The consumption of 2,3-diphosphoglycerate in stored red blood cells may change the oxygen dissociation curve and may impair oxygen delivery [44]. In addition, mechanical damage and turbulent channels in CPB may enhance hemolysis. Hemolysis of red blood cells increases the concentration of free hemoglobin and iron, as well as pro-inflammatory molecules, which are toxic to the kidneys. Our findings confirmed that with the increase of intraoperative blood transfusion, the incidence of postoperative AKI also increases.

Furthermore, our study confirmed that the risk of AKI after surgery increased with an increase in the CPB time, which was similar to that reported in previous studies [45]. AKI may be related to renal hypoperfusion and ischemia reperfusion injury caused by CPB [46, 47]. Moreover, CPB stimulates the systemic inflammatory response, leading to release of a series of pro-inflammatory mediators and factors, which further causes AKI [48, 49]. Therefore, based on current clinical evidence, reducing the CPB time to the maximum possible extent remains the focus to decrease the risk of postoperative AKI.

SBS is widely used considering its theoretically protective effects on renal tubules. A double-blind, randomized, controlled trial that recruited many patients who had undergone cardiac surgery proposed that perioperative urinary alkalization reduced the incidence of postoperative AKI from 52 to 32%, with no apparent side effects [50]. The mechanism related to this effect is thought to be related to the ability of bicarbonate to alkalize urine and slow-down the Haber-Weiss reaction that produces active oxygen through iron-dependent pathways [51]. Moreover, another mechanism is that bicarbonate can directly remove other reactive substances present in blood, including hydroxyl radicals and peroxynitrite [52]. However, the protective role of alkalization against AKI has received widespread attention in recent years and has also raised queries. Recent studies have suggested that infusion of SBS does not reduce the incidence of AKI after open-heart surgery [53]. In 2007, a study by Haase et al. showed that sodium bicarbonate is an effective, simple, practical, and inexpensive treatment that can prevent the toxic effects of hemolysis on the kidneys during CPB [50]. Whereas, they reached conflicting conclusions through a multi-center double-blind randomized controlled experiment in 2013. They proposed that in high-risk patients with AKI after open-heart surgery, bicarbonate infusion can alkalinize blood and urine, but cannot reduce the incidence of AKI or reduce acute tubular injury. Importantly, prophylactic use of sodium bicarbonate injection may increase mortality [54]. Our study supports the aforementioned view. We found that increased intraoperative infusion of 5% SBS was associated with higher incidence of AKI after surgery.

Urine volume is closely related to renal function and has been used as a diagnostic criterion for AKI. It has been clinically recognized that insufficient renal perfusion is the main factor that promotes AKI after cardiac surgery [55]. Song et al. divided the patients into two subgroups according to the urine output of less than or more than 4 mL/kg/h during CPB. The urine output was determined to be an independent predictor of AKI, with odds ratios of 0.43 and 1.11, respectively [56]. They believe that urine output can be used as a simple and feasible method to early predict the development of AKI after cardiac surgery. Yilmaz M et al. also believe that urine output during CPB is an important criterion for predicting AKI after coronary artery bypass graft surgery [57]. However, some scholars have proposed that the damage of renal tubular reabsorption and the heterogeneity of nephron function may contradictorily increase the urine output, and maintaining the urine flow may not guarantee the normal function of the kidney. In the same context, excessive urination during CPB should not be interpreted as a favorable signal, as renal tubular damage caused by inflammation and thrombosis during CPB may increase urine flow. Our findings believe that intraoperative urine output is a friendly factor for postoperative AKI. More targeted research should be conducted to understand the relationship between intraoperative urine volume and postoperative AKI in patients undergoing CPB cardiac surgery.

## Conclusion

The prediction of postoperative AKI in patients undergoing cardiac surgery with CPB is critical as the complication can affect the overall survival of patients. A novel nomogram was developed and its application was reported in this study. The nomogram demonstrated relatively good accuracy to help clinicians understand the risk of postoperative AKI in patients undergoing cardiac surgery with CPB, thereby enabling appropriate measures to be taken for early medical intervention.

## Supplementary Information

**Additional file 1**.

## Data Availability

The release of data in this study did not involve the identity information of patients. The full text does not contain any direct or indirect identifiers that expose the basic information of patients, and the clinical information of all participants is completely anonymous. All data generated during this study are included in this published article [and its supplementary information files].
